# Single molecule measurements of microRNAs in the serum of patients with pulmonary tuberculosis

**DOI:** 10.3389/fimmu.2024.1418085

**Published:** 2024-09-02

**Authors:** Zhuhua Wu, Qiuchan Tan, Xiaojuan Jia, Huizhong Wu, Jing Liang, Wenpei Wen, Xuezhi Wang, Chenchen Zhang, Yuchuan Zhao, Yuhui Chen, Tingrong Luo, Wenjun Liu, Xunxun Chen

**Affiliations:** ^1^ College of Animal Science and Technology, Guangxi University, Nanning, Guangxi, China; ^2^ Key Laboratory of Translational Medicine of Guangdong, Center for Tuberculosis Control of Guangdong Province, Guangzhou, Guangdong, China; ^3^ School of Basic Medical Sciences, Guangzhou Health Science College, Guangzhou, Guangdong, China; ^4^ CAS Key Laboratory of Pathogenic Microbiology and Immunology, Institute of Microbiology, Chinese Academy of Sciences, Beijing, China; ^5^ Dongguan Key Laboratory of Tuberculosis Control, The Sixth People’s Hospital of Dongguan, Dongguan, Guangdong, China; ^6^ Department of Laboratory Medicine, Foshan Fourth People’s Hospital, Foshan, Guangdong, China; ^7^ State Key Laboratory for Conservation and Utilization of Subtropical Agro-Bioresourses & Laboratory of Animal Infectious Diseases, College of Animal Sciences and Veterinary Medicine, Guangxi University, Nanning, Guangxi, China

**Keywords:** tuberculosis, microRNAs, single molecule array, biomarker, Serum

## Abstract

**Background:**

microRNAs (miRNAs) were recognized as a promising source of diagnostic biomarker. Herein, we aim to evaluate the performance of an ultrasensitive method for detecting serum miRNAs using single molecule arrays (Simoa).

**Methods:**

In this study, candidate miRNAs were trained and tested by RT-qPCR in a cohort of PTB patients. Besides that, ultrasensitive serum miRNA detection were developed using the Single Molecule Array (Simoa) platform. In this ultra-sensitive sandwich assay, two target-specific LNA-modified oligonucleotide probes can be simply designed to be complementary to the half-sequence of the target miRNA respectively. We characterized its analytical performance and measured miRNAs in the serum of patients with pulmonary tuberculosis and healthy individuals.

**Results:**

We identified a five signature including three upregulated (miR-101, miR-196b, miR-29a) and two downregulated (miR-320b, miR-99b) miRNAs for distinguishing PTB patients from HCs, and validated in our 104 PTB patients. On the basis of Simoa technology, we developed a novel, fully automated digital analyser, which can be used to directly detect miRNAs in serum samples without pre-amplification. We successfully detected miRNAs at femtomolar concentrations (with limits of detection [LODs] ranging from 0.449 to 1.889 fM). Simoa-determined serum miR-29a and miR-99b concentrations in patients with PTB ((median 6.06 fM [range 0.00–75.22]), (median 2.53 fM [range 0.00–24.95]), respectively) were significantly higher than those in HCs ((median 2.42 fM [range 0.00–28.64]) (*P < 0.05*), (median 0.54 fM [range 0.00–9.12] (*P < 0.0001*), respectively). Serum levels of miR-320b were significantly reduced in patients with PTB (median 2.11 fM [range 0.00–39.30]) compared with those in the HCs (median 4.76 fM [range 0.00–25.10]) (*P <* 0.001). A combination of three miRNAs (miR-29a, miR-99b, and miR-320b) exhibited a good capacity to distinguish PTB from HCs, with an area under the curve (AUC) of 0.818 (sensitivity: 83.9%; specificity: 79.7%).

**Conclusions:**

This study benchmarks the role of Simoa as a promising tool for monitoring miRNAs in serum and offers considerable potential as a non-invasive platform for the early diagnosis of PTB.

## Introduction

Tuberculosis (TB) remains a major public health problem worldwide. According to the WHO Global TB Report 2023, an estimated 10.6 million people fell ill with TB and about 1.3 million people died from it in 2022 (1). Early, rapid, and accurate diagnosis has been recognised as a pillar to achieving an end TB strategy (2). Therefore, it is crucial to identify novel biomarkers for TB to develop a practical method for diagnosis.

MicroRNAs (miRNAs) are highly conserved, short, non-coding RNAs that guide post-transcriptional gene regulation and involved in shaping immunity (3). Beyond their expression in specific tissue, miRNAs are also present in different body fluids (4). With their relatively stable, reproducible during freezing thawing and extended storage, substantial levels of sensitivity, miRNAs are considered to be promising diagnostic biomarkers (5). By 2024, numerous studies reported blood miRNA expression profiles in patients with pulmonary tuberculosis (PTB) and healthy individuals, indicating that miRNAs play a vital role in post-transcriptional regulation in the pathogenesis of *Mycobacterium tuberculosis* (Mtb) and have the potential to be diagnostic and prognostic non-invasive biomarkers (6, 7). However, there are inconsistencies in the data reported across all studies and overlapping expression patterns were rarely found. Whether this is due to the sequencing platform, small sample size and the screening criteria for candidates remains to be clarified.

Despite miRNAs have been scrutinized as prospective biomarkers, conventional miRNAs detection methods have not yet been performed in clinical routine due to their own limitations. Serum, plasma or whole peripheral blood is routinely collected and relatively non-invasive to sample, can be used as starting material. However, in many cases, isolation of miRNAs requires additional extraction and purification steps. In addition, due to extremely low levels of miRNAs, it is necessary to develop new detection technology with high sensitivity for low content miRNAs in complex biological clinical samples. Currently, conventional miRNA detection methods, including quantitative real-time polymerase chain reaction (RT-qPCR), next-generation sequencing (NGS), northern blot-based platforms, *in situ* hybridisation, and microarray, are far from ideal diagnostic tools for routine clinical use (8, 9). Recent studies reported various novel miRNA detection methods based on signal amplification and discrimination strategies (10). However, the performance of these new methods needs to be evaluated and optimised.

Single molecule array (Simoa) is an ultrasensitive technique capable of detecting single biomolecules (11). By utilizing Simoa, protein biomarkers as well as DNA with very low expression levels could be detected in many diseases, but few studies have been reported on miRNA detection (12–14). Simoa was first used for miRNA detection by Cohen et al. in 2017 (15). Simoa has the advantages of high sensitivity (with LODs ranging from 1 to 30 fM) and minimal processing steps (no additional extraction and purification steps required) and allows quantitative measurement of miRNAs without pre-amplification; it can be used to detect various disease-related miRNAs in real-time and has potential value in clinical practice.

In this study, we focused on assessing miRNA expression in the serum of patients with PTB in comparison to that in healthy controls (HCs) using Simoa. We performed an integrated miRNA-TB expression profiling analysis to screen out the miRNAs reported in at least three studies, these candidate miRNAs were further verified by RT-qPCR in a cohort of PTB patients. Besides that, we directly detects miRNAs without pre-amplification using Simoa, and apply it to the detection of miRNAs in serum samples from PTB and healthy individuals, and a potential diagnostic model was established by logistic regression.

## Materials and methods

### Selection of studies and datasets

A systematic literature review was performed to identify miRNA expression profiling datasets comparing patients with PTB and HCs. We conducted a systematic search of the PubMed databases, using the MESH search headings: (“Tuberculosis”[Mesh]) AND “MicroRNAs”[Mesh]. Reviewers independently screened titles and abstracts, relevant articles were retrieved and assessed independently, and extracted the following data including: first author, year of publication, country, platform, no. of samples, miRs validated, No. of qRT-PCR validated sample (TB/HC) and the list of up- and down-regulated miRNAs features, cut-off criteria and their relative fold change. Vote-counting rank was used to rank potential molecular biomarkers according to their importance as follows: (a) number of comparisons reported; (b) total number of samples for comparison in agreement; (c) average fold-changes reported for comparisons in agreement. Total number of samples was considered more important than average fold-change because many studies did not report a fold-change. For more information on the studies and dataset selection, see the **Supplementary Material**.

### Ethical statement

This study was approved by the Center for Tuberculosis Control of Guangdong Province Ethics Committee, Guangzhou, China. Written informed consent was obtained from all participants prior to their enrolment in the study.

### Sample collection

Patients were recruited and enrolled at the outpatient department and health examination centres of the Center for Tuberculosis Control of Guangdong Province, Guangzhou, China. The demographic and clinical characteristics of the PTB and HC groups are summarised in **Table 1**. PTB patients diagnosed based on clinical signs, chest radiography, and smear microscopy. Participants who had another coexisting disease were excluded. Five millilitres of peripheral venous blood was extracted using a sterile polyolefin resin tube without anticoagulant. Serum samples were stored at -80°C until RNA was extracted.

**Table 1:** Characteristics of study participants with PTB and HCs.

Variable	Screening set (N=36)			Validation set (N=205)			Sioma assay (N=121)		
	PTB	HCs	P valve	PTB	HCs	P valve	PTB	HCs	P valve
N	18	18		104	101		62	59	
Gender									
Male	11	8	0.317	54	42	0.138	31	27	0.641
Female	7	10		50	59		31	32	
Age, yr	31.56 ± 8.05	33.83 ± 9.48	0.442	32.12 ± 9.99	34.28 ± 9.40	0.114	34.6 ± 11.44	33.41 ± 9.65	0.538
Radiographic features of PTB	18	0		104	0		62	0	
Smear microscopy-positive TB	4	0		17	0		12	0	
IGRA									
Positive	NA	0		NA	0		NA	0	
Negative	NA	18		NA	101		NA	59	

“NA”, Not available

### Total RNA extraction and RT-qPCR

Total miRNAs were isolated from serum using the miRNeasy Serum/Plasma Kit (Qiagen, Germany) according to the manufacturer’s instructions. The miRNAs were reverse-transcribed to cDNA using a TaqMan™ MicroRNA Reverse Transcription Kit (Applied Biosystem, USA). Thereafter, the quantification of miRNAs was performed using the HieffTM qPCR SYBR Green Master Mix (Vazyme, China) on a ViiA7 instrument. Ce_miR-39 was used as the reference gene. Relative fold changes of miRNA expression were calculated using the ΔΔCT method. RT-specific stem-loop primers and the miRNA-specific forward primer sequences used in this study are listed in **Supplementary Table S1**.

### Probe design

Locked nucleic acid (LNA) capture and detection probes were designed as previously described (16). LNA-modified capture and detection probes with sequences complementary to 11 or 12 bases of target miRNAs were used to increase hybridization specificity. The placement of bases within each LNA-modified capture

probes and detection probes were then adjusted according to the following criteria: (1) consistent melting temperature; (2) strong predicted binding between each probe and the target sequence; (3) low cross-reactivity. Specially, the capture probe was modified using 5’-Amino-Modifier C12 and detection probe was biotinylated on the 3’ end. The capture and detection probes used in this study are listed in **Supplementary Table S2**.

### Affinity measurements between probes and target miRNAs

Affinity measurements was conducted as described previously (15). Capture probes, detection probes, and synthetic target miRNAs were synthesized from TaKaRa Biotechnology (Dalian, China). Affinity measurements were performed using the NeoSPR-M100 System (Neoline, China) to explore the potential interactions between the probes and target miRNAs.

### Simoa assays

miRNA Simoa assays were performed according to previously published procedures with some modifications (15). Briefly, the microbeads were diluted in hybridisation buffer to a concentration of 2 × 10^7^ beads/mL after covalent coupling of the capture probes to paramagnetic microbeads. Samples and capture beads were placed in a 96-well conical bottom microtiter plate (Quanterix, USA) and incubated at 30°C for 60 min. Biotinylated detection probes were added after cleaning the capture beads. After incubation with specific biotinylated detection probes, streptavidin-β-galactosidase was added and incubated at 30°C with shaking for 20 min. Resorufin β-D-galactopyranoside and Simoa Sealing Oil were loaded onto the Simoa HD-1 Analyser (Quanterix, USA) according to the manufacturer’s instructions. The software on the HD-1 Analyser was used to determine the average number of enzymes per bead (AEB). The software was also used to interpolate the concentration of miRNAs in the samples from a calibration curve.

### Statistical analyses

Calibration curves were fitted to a 4-parameter nonlinear regression model with a 1/Y2 weighting factor. SPSS software (version 16.0) was used for statistical analyses. Data from each group are presented as the mean ± standard deviation (SD). The Mann–Whitney U test (nonparametric) was used to compare the differences in serum miRNA concentrations among different groups. Statistical significance was set at *P <*0.05.

## Results

### Characteristics of study participants

A total of 362 participants, including 184 patients with PTB and 178 HCs, were enrolled in the study. Patients with PTB and the HCs were divided into three phases: Screening set (n = 36), Validation set (n = 205), and Simoa assay phase (n = 121). The demographics and clinical parameters of the PTB and HC samples are listed in **Table 1**. No significant differences (*P >*0.05) were found in the distribution of sex and age among the patients with PTB and the HC samples.

### Screening and overview of datasets

**Figure 1:**
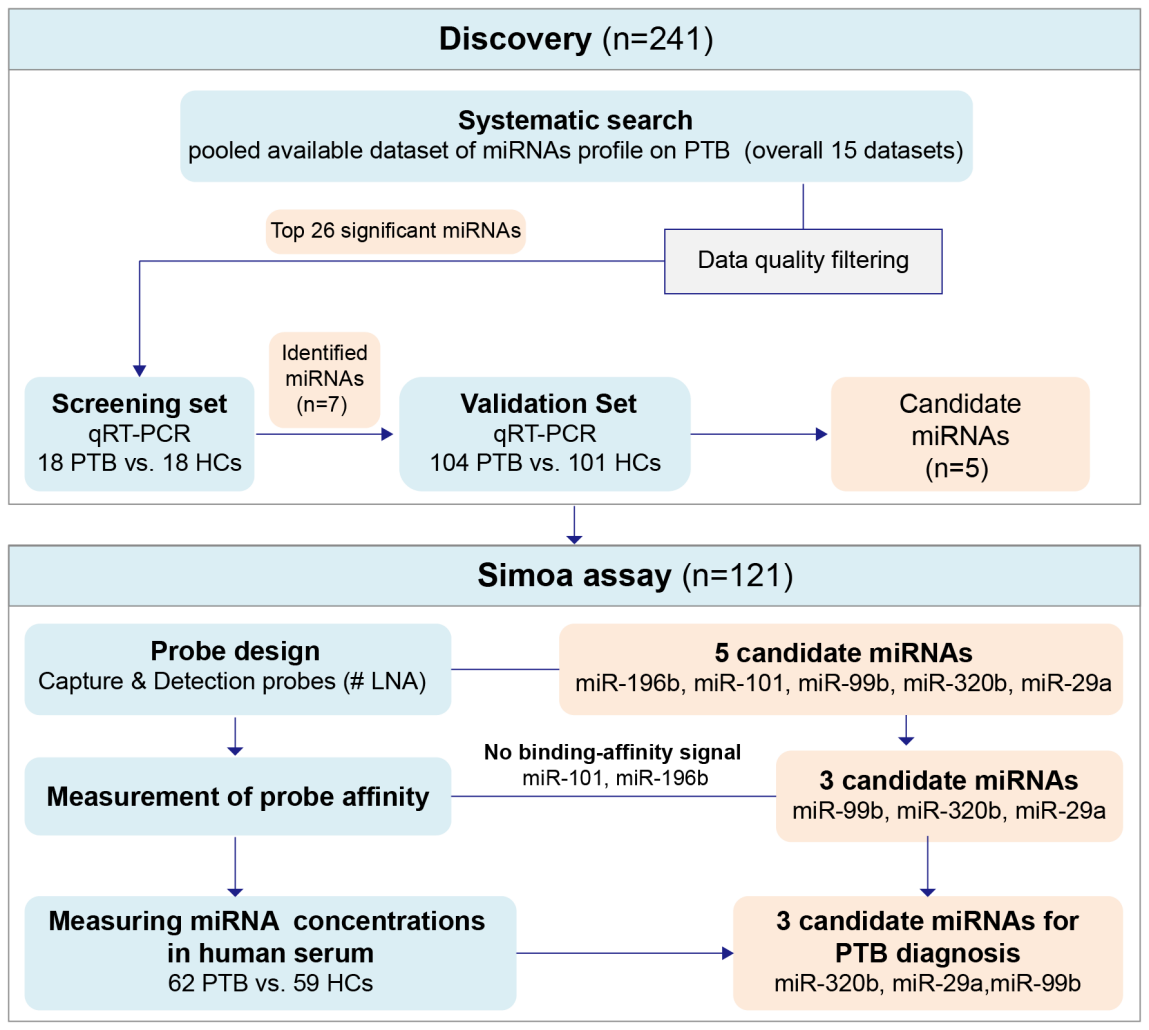
The flowchart of the experiment design strategy. Discovery phase: to discover and preliminarily validate the serum miRNAs differentially expressed between PTB patients and HCs, the signatures screened in the miRNAs expression profile were tested in the testing set and then applied in the external testing cohort. Simoa assay phase: to further validate the selected differentially expressed candidate miRNAs. HCs, Healthy controls; PTB, pulmonary tuberculosis.

A flowchart of the experimental design strategy is shown in **Figure 1**. Fifteen TB miRNA expression profiles were identified in this integrated miRNA-PTB expression profiling analysis (**Supplementary Figure S1**). The characteristics of each dataset are presented in **Supplementary Table S3**. Sixty-three differentially expressed miRNAs were identified in the fifteen miRNA expression profile datasets, of which fifty-two were upregulated and eleven were downregulated in at least three studies (**Supplementary Table S4**). Heatmap analysis showed that some miRNAs were inconsistently upregulated or downregulated (**Supplementary Figure S2A**). Following profiling, 12 of the 15 studies performed RT-qPCR validation of the selected 80 miRNAs. Among those 80 miRNAs, 26 were significantly (*P <*0.05) upregulated, and 4 were significantly (*P <*0.05) downregulated (**Supplementary Figure S2B**, **Supplementary Table S5**).

### Confirmation of identified miRNAs via RT-qPCR

**Figure 2:**
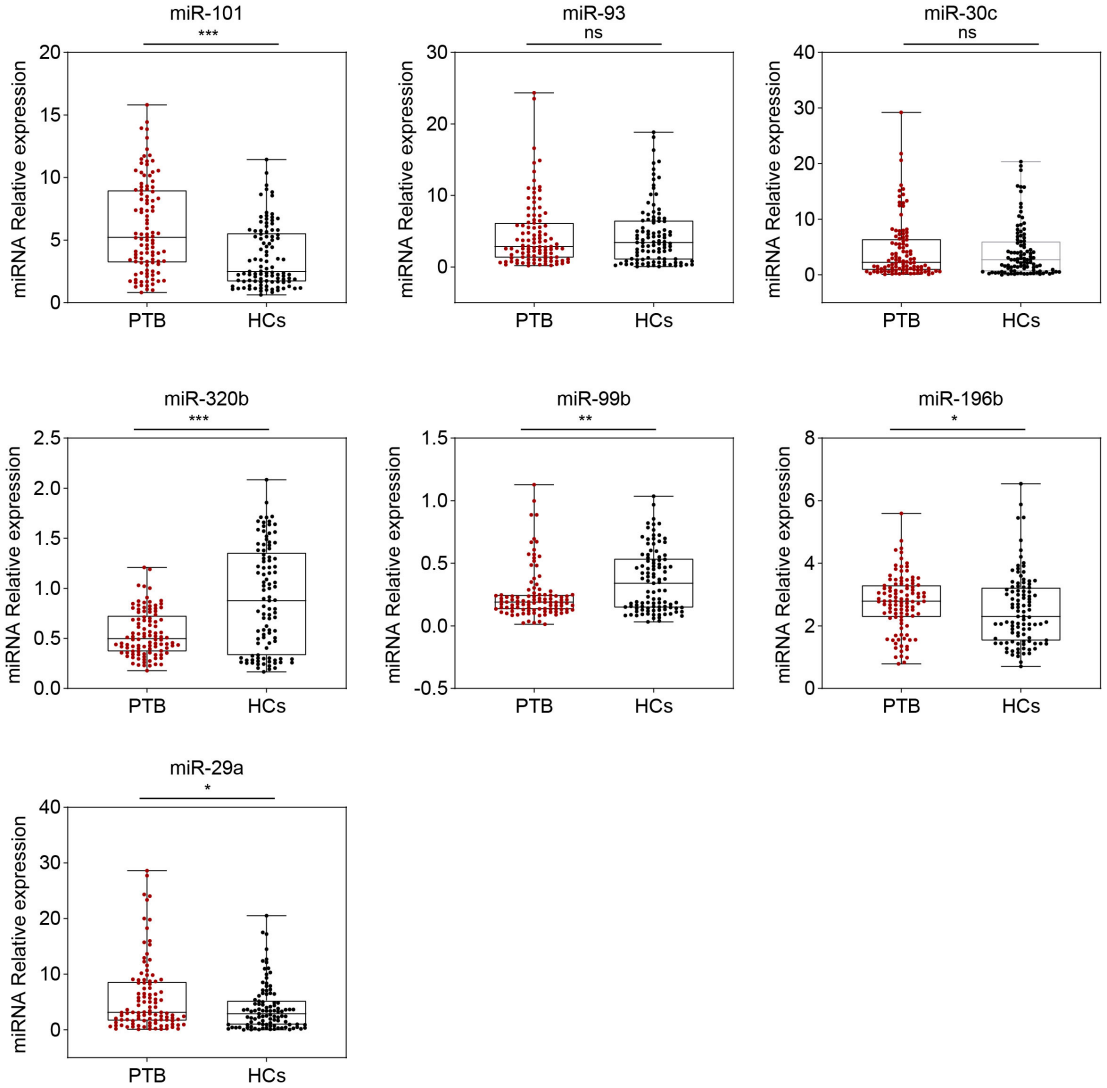
Seven serum miRNA levels in patients with PTB (n = 104) and HCs (n = 101) were selected for verification using the RT-qPCR (in the validation set). The relative abundance of the miRNAs is represented in boxplots. ns, no significance, *P <0.05, **P <0.01, ***P <0.001.

Based on the miRNA-PTB expression profiling analysis of the miRNA screening results, we selected the top 26 significant miRNAs for further examination of PTB-associated miRNA expression using RT-qPCR. Seven miRNAs were differentially expressed in the screening set (fold change >2, *P <*0.05) (**Supplementary Table S6**) and were subsequently validated in the validation set, which included 104 patients with PTB and 101 HCs. The data showed that five miRNAs (miR-101, miR-320b, miR-99b, miR-196b, and miR-29a) were significantly differentially expressed between the PTB and HC groups (*P <*0.05) (**Figure 2**).

### Determination of binding affinity of probes to target miRNAs

**Figure 3:**
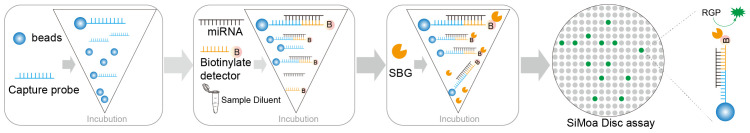
Overview of Sioma detection and probe affinity analysis. Schematic of the assay for miRNA detection using Simoa.

The objective of this study was to develop a Simoa assay to detect miRNAs directly in serum without nucleic acid purification and pre-amplification. Serum samples are incubated with paramagnetic microbeads coated with capture probes. The bead-miRNAs complexes are incubated sequentially with the biotinylated detection probes, SBG and loaded to the Simoa disc array. **Figure 3** shows a schematic of the detection of miRNA using Simoa by developing a bead-based sandwich protocol.

**Figure 4:**
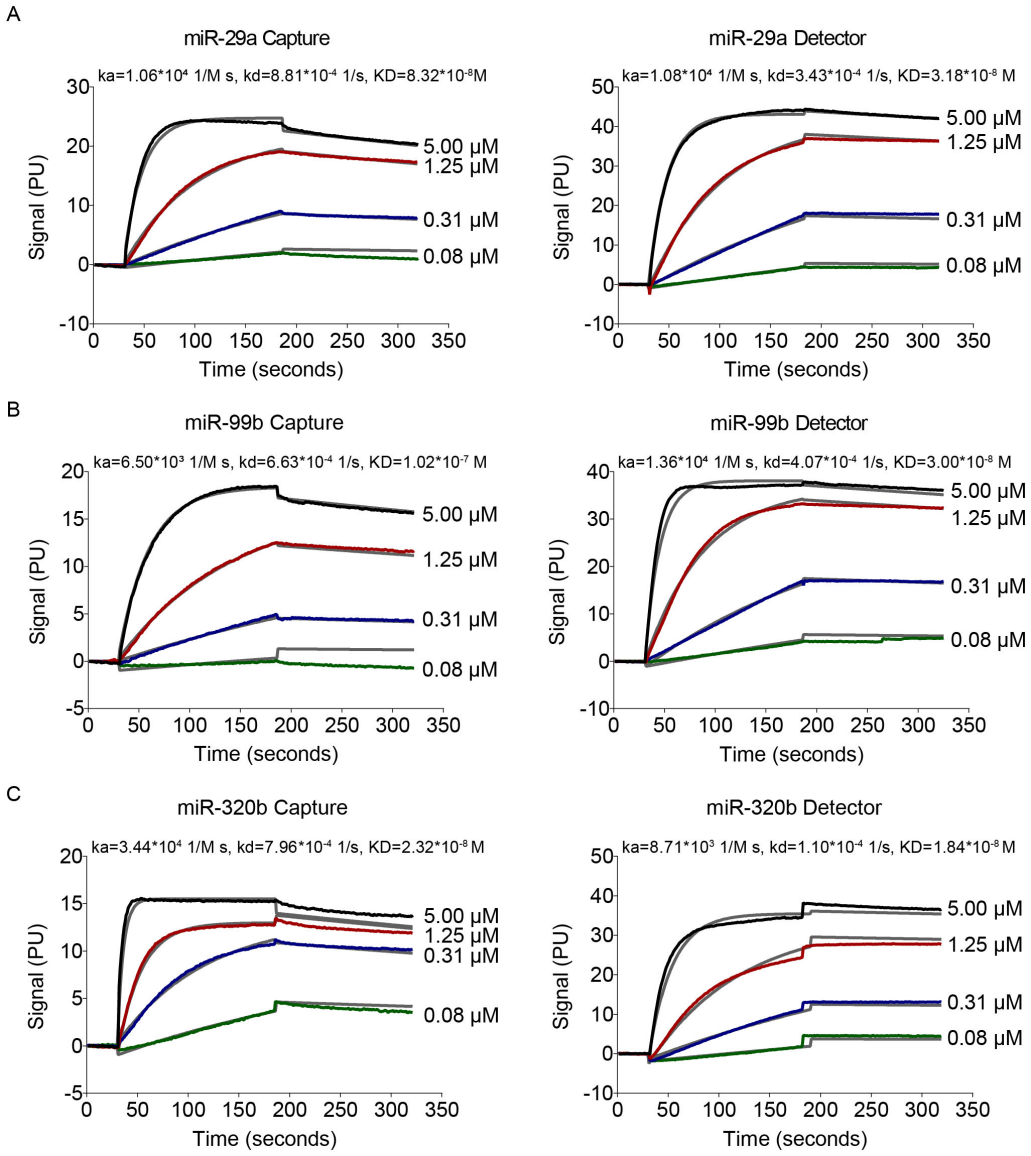
Data of affinity interactions between capture probes to target miRNA or detection probes to target miRNA. The binding affinity was determined using 4-fold serial dilutions at concentrations of 0.08–5 μM.

To examine the hybridisation efficiency, we examined the affinity of capture and detection probes for target miRNAs by the NeoSPR-M100 System. After the raw data were analysed, the K_D_ values of capture and detection probes for miR-29a were 8.32 ×10^-8^ and 8.18×10^-8^ M, respectively (**Figure 4A**); The K_D_ values of capture and detection probes for miR-99b were 1.07 ×10^-7^ and 3.00×10^-8^ M, respectively (**Figure 4B**); The K_D_ values of capture and detection probes for miR-320b were 2.32 ×10^-8^ and 1.84×10^-8^ M, respectively (**Figure 4C**). Values of K_D_ shown in **Figure 4** indicate that the affinity of the capture/detection probes for the target miRNAs increased with increasing concentration. No binding-affinity signal was detected for miR-101 and miR-196b.

### Detection of miRNA concentrations in serum samples using Simoa

**Figure 5:**
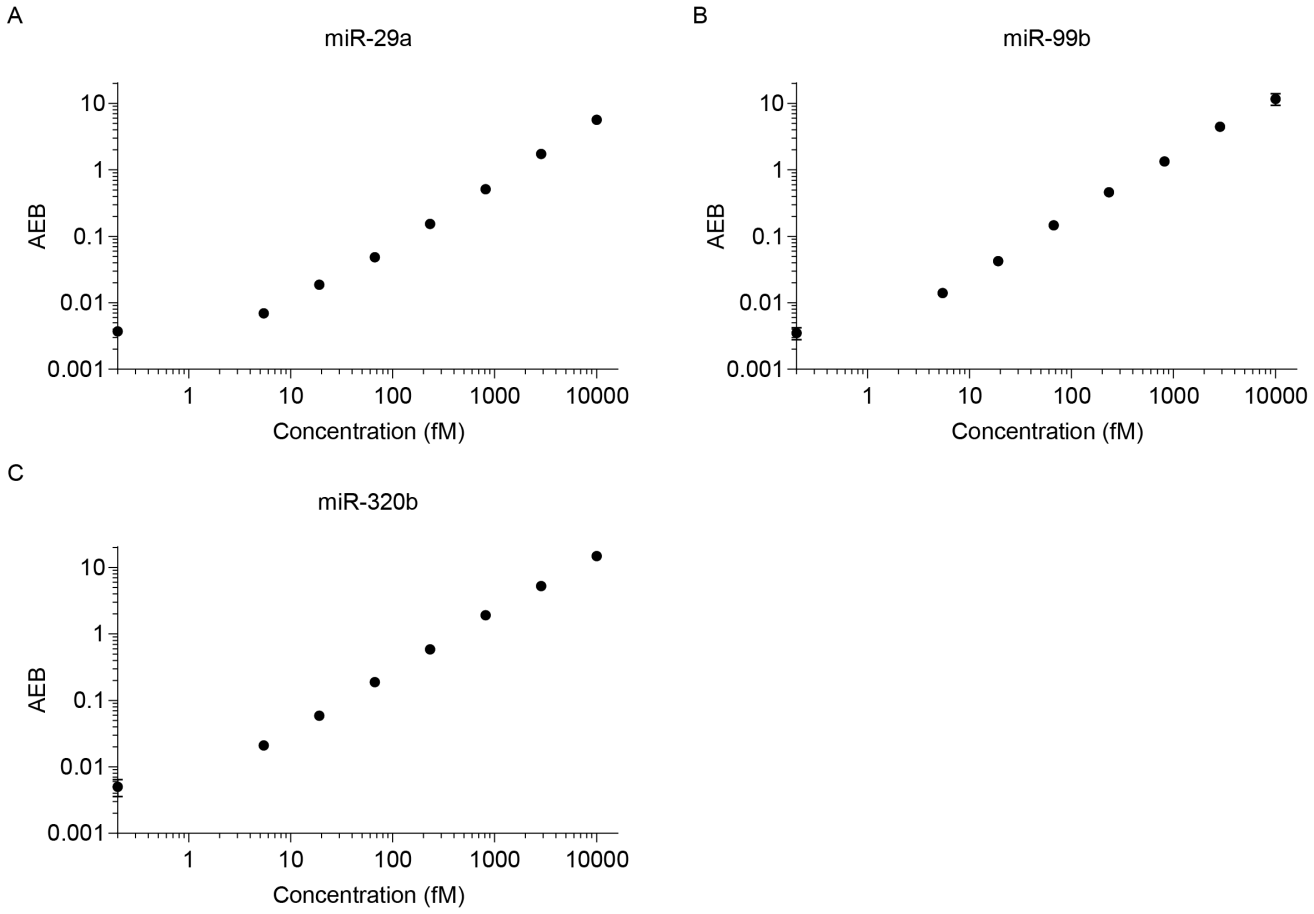
Calibration curves for various miRNAs. LODs were calculated as three standard deviations: **(A)** miR-29a (LOD 1.889 fM), **(B)** miR-99b (LOD 0.449 fM), and **(C)** miR-320b (LOD 0.462 fM).

**Figure 5** shows the AEB values for buffered solutions, into which known concentrations of a synthetic calibrator for each target miRNA were spiked, ranging from 0 to 10000 fM. All assays yielded similar results, with LOD of miR-99b, miR-320b, and miR-29a at 0.449, 0.462, and 1.889 fM, respectively (**Figure 5**). To demonstrate the potential use of the Simoa assay for clinical testing, we developed assays for the detection of miRNAs in human serum without a separate isolation step.

**Figure 6:**
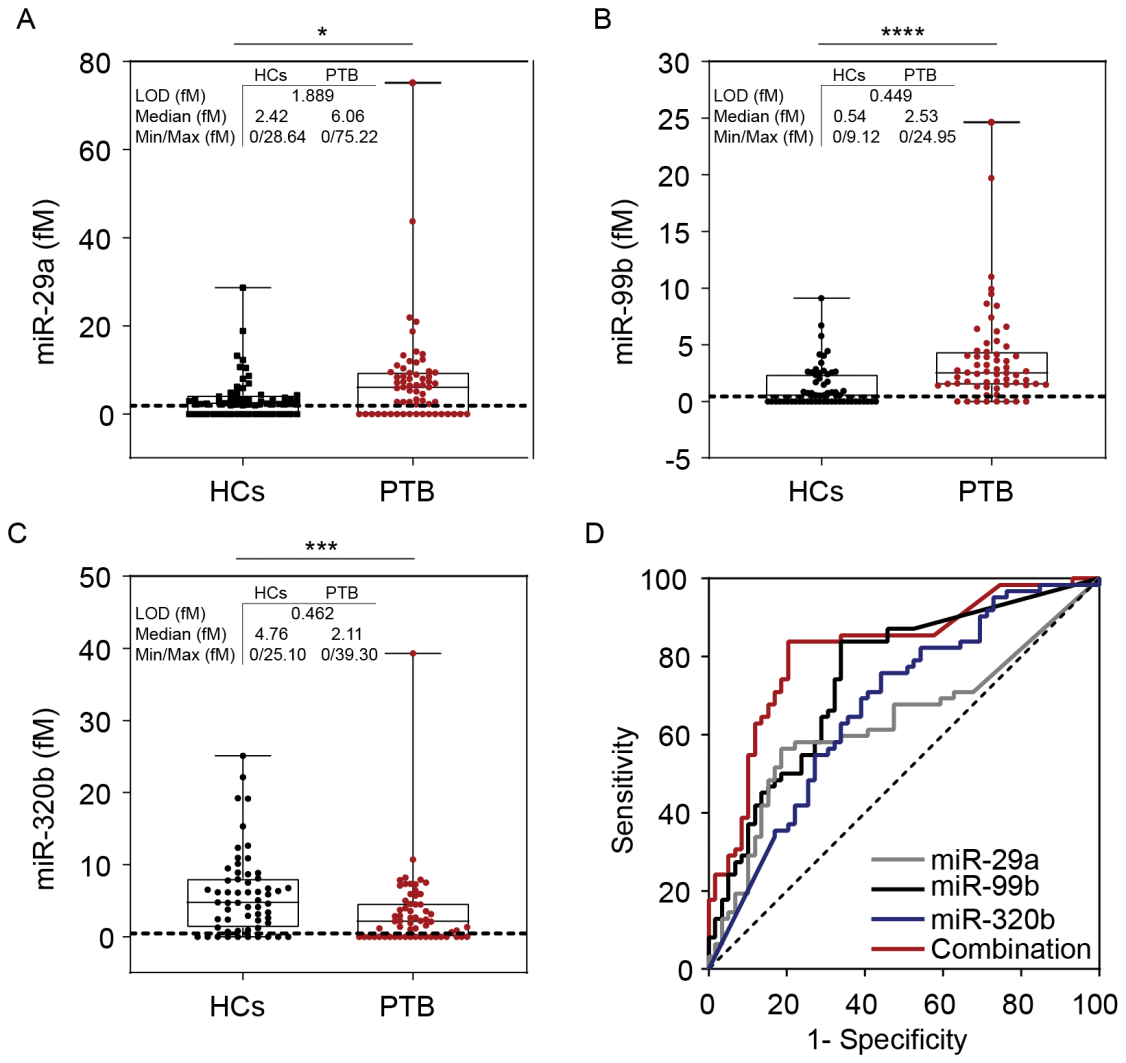
Simoa assay for miR-29a, miR-99b, and miR-320b. Direct detection of **(A)** miR-99b, **(B)** miR-29a, and **(C)** miR-320b concentrations in the serum of patients with PTB (n = 62) and HCs (n = 59). Dashed lines indicate LOD values. The concentration of the miRNAs is represented in boxplots. **(D)** The ROC curve of miR-29a, miR-99b, miR-320b, and the combined model were analysed. LOD, limit of detection. *P <0.05, ***P <0.001, ****P <0.0001.

Simoa-determined serum miR-29a concentrations in patients with PTB ((median 6.06 fM [range 0.00–75.22]) were significantly higher than those in HCs (median 2.42 fM [range 0.00–28.64]) (*P* < 0.05) (**Figure 6A**). The miR-99b concentrations also significantly differed between patients with PTB (median 2.53 fM [range 0.00–24.95]) and the HCs (median 0.54 fM [range 0.00–9.12] (*P < 0.0001*) (**Figure 6B**). Serum levels of miR-320b were significantly reduced in patients with PTB (median 2.11 fM [range 0.00–39.30]) compared with those in the HCs (median 4.76 fM [range 0.00–25.10]) (*P <* 0.001) (**Figure 6C**).

Receiver operating characteristic curve (ROC) curve analyses were conducted to assess the diagnostic value for discriminating between patients with PTB and HCs, yielding the following AUCs: miR-29a, 0.633 (P <0.05, 95%CI: 0.531–0.734); miR-99b, 0.748 (P <0.0001, 95%CI: 0.661–0.834); miR-320b, 0.675 (P <0.01, 95%CI: 0.579–0.771). We performed a risk score analysis on the combined cohorts and found that the 3-miRNA panel had a greater separating capacity than one particular miRNA, as evidenced by an AUC of 0.818 (P <0.0001, 95% CI: 0.741–0.896). The sensitivity was 83.9% and the specificity was 79.7%, which reflected reasonable separation between the two groups (**Figure 6D**).

## Discussion

In the present study, we performed an integrated miRNA-PTB expression profiling analysis to screen out miRNAs reported in at least three studies that were trained and tested using RT-qPCR in a cohort of patients with PTB. We also evaluated the diagnostic accuracy of these candidate miRNAs as PTB biomarkers. In addition, we developed an ultrasensitive and non-invasive serum miRNA detection assay using capture and detection probes with fully automated Simoa technology. Our work systematically evaluated candidate miRNAs as diagnostic markers and indicated that Simoa shows promise for the ultrasensitive detection of miRNAs in human serum samples. This assay offers high-sensitivity, flexibility and affordability. The platform has a high degree of automation, all the measurements and exercise protocols can be performed in approximately 5 hours. Furthermore, Simoa technology allows for multiplexed detection, and from a reagent standpoint, the cost/test for the miRNAs assay would be expected to be comparable to conventional TB immunoassays due to our use of low cost bead-based reagents and commercially available probes.

Monumental studies have reported blood miRNA expression profiles in patients with PTB. However, there is a lack of consensus on miRNA panels for detecting PTB development. Summary and integration analysis of raw expression datasets is the preferred method for miRNA expression analysis; however, this rigorous approach is difficult to achieve because of the unavailability of raw data. Of the reported studies, 11 out of 15 conducted a preliminary analysis to evaluate miRNA expression in their cohorts using microarray or sequencing. Only Maertzdorf’s (17) and Zhang’s (18) published raw data on public data sources. Considering the inconsistent annotation and discovery of new miRNAs, we attempted to find a simple and effective way to improve the statistical power of microarray and sequencing data by integrating the results of each study. Hence, we performed an integrated miRNA-PTB expression profiling analysis to identify miRNAs reported in at least three studies. Our integrated miRNA-PTB expression profiling analysis distinguished significantly upregulated and downregulated miRNAs in patients with PTB compared with those in HCs; however, there were inconsistencies in the reported data, and overlapping expression patterns were rarely found.

For example, Zhang et al. (19) and Tu et al. (20) reported that miR-320b was downregulated in serum samples from patients with PTB. In contrast, miR-320b was upregulated in a study by Wang et al. (21, 22). According to our RT-qPCR validation, miR-320b was downregulated in patients with PTB compared with that in HCs. A systematic review of miRNAs as biomarkers in TB revealed that miR-320b was downregulated in patients with PTB compared with that in HCs, which is consistent with our RT-qPCR verification and Simoa detection results (23). In addition, four studies showed inconsistent results; two studies reported that miR-99b was upregulated in serum samples from patients with PTB (21, 22), whereas Wang et al. (18, 24) found that miR-99b expression was downregulated. Similarly, the RT-qPCR and Simoa assays conducted in this study reported inconsistent expression trends, and the RT-qPCR results did not coincide well with the Simoa measurements for both relative and absolute quantification. Previous studies have demonstrated that miR-99b is highly expressed in Mtb-infected dendritic cells, suggesting its participation in immune evasion (25) and suppression of inflammation (26); however, neutrophil-associated miR-99b transcript levels are significantly low in TB cases (27). Despite the heterogeneity of the results, some miRNAs have been identified as candidate biomarkers for TB in more than one study. A study on plasma used miRNA PCR panels to obtain a short-list of candidates that were validated via RT-qPCR. Notably, two miRNAs (miR-99b and miR-29a) with high expression in plasma were upregulated in patients with PTB compared with those in the controls (28). The remaining two miRNAs (miR-29a and miR-196b) showed consistent expression trends in serum samples (17, 18, 24, 29, 30). miR-29a and miR-196b levels were elevated in the serum and plasma samples of patients with TB, which is consistent with our RT-qPCR results. According to a new study, there was a statistically significant decrease in the expression of miR-454-3p, miR-15a-5p, miR-590-5p, miR-381, and miR-449a in the Pulmonary TB group compared with those in the healthy control group (31).

At present, northern blotting, microarray and qRT-PCR are conventional methods for miRNAs detection, however, they are not capable of detecting miRNAs in complex biological clinical samples and do not provide good performance in ultra-sensitive miRNAs detection (9). The Simoa technique has been previously utilized for ultra-sensitive detection of proteins (32), since the platform allows detection of sufficiently small particles, it has also been used to detect DNA (33) or miRNAs (12). Thus, we have developed an ultrasensitive and non-invasive serum miRNAs detection assay using capture and detector probes with fully automated Simoa technology. In the present study, we focused on the development and optimisation of efficient methods for miRNA detection in serum samples using the Simoa platform. In this process, it is crucial to design LNA-modified capture and detection probes. Both these probes must bind to the target miRNAs and generate signals. The results indicated that the target miRNAs had strong binding abilities with the capture/detection probes. Unfortunately, miR-101 and miR-196b was excluded from further Simoa detection because a binding affinity signal was not detected for it. We used the Simoa platform to measure miRNAs in the serum without a separate separation step. For any potential future clinical application, it is notable that all serum samples were well in the measurable range of the Simoa assay. We found that the Simoa platform exhibits limits of detection in the range of 0.449-1.889 fM. The WHO has included the development of diagnostic biomarkers in its high-priority target product profile (TPP), (non-sputum-based diagnostic: >90% sensitivity and >70% specificity) (34). For active case finding, Simoa detection did not meet TPP benchmarks for a triage test. However, our data demonstrates the moderate sensitivity and specificity offered by a combination of three miRNAs detection in distinguishing PTB from HCs. We are confident that the Simoa assay can

serve as a valuable tool for TB screening. There is a growing tendency to integrate multiple miRNAs into a biomarker panel, which can enhance both sensitivity and specificity. The composite expression profile of several miRNAs can furnish a more refined biomarker signature, enhancing the discriminative capability between PTB and HCs. Moreover, miRNA expression can vary with disease progression, suggesting the value of dynamic monitoring rather than relying solely on static levels. As the protocol for the Simoa assay is still under development, there are some limitations to our study. The detection sensitivity needs to be further optimised. The percentage of all serum samples for whom the miRNAs were consistently >LOD in any measurement was 69.4% (88/121) for miR-29a, 70.2% (85/121) for miR-99b, and 74.4% (90/121) for miR-320b. Secondly, because these miRNAs can be similarly expressed in other infections of the lungs, such as bacterial pneumonia or NTM infection. Although statistically significant differences in miRNA expression have been identified, translating these findings into clinical practice requires rigorous validation. To enhance the reliability, quality assurance, and validity of these biomarkers, it is essential to establish standardized procedures for the preparation of various sample types. Furthermore, the development of better methods to assess the quality of miRNA markers is critical. Therefore, in subsequent studies, we will optimise the sensitivity of Simoa assays by maximising the capture of target molecules and subsequently use the minimum concentrations of labelling reagents to yield the highest signal-to-background ratio. In addition, expanding the patients cohort for further validation is necessary in order to test fully the utility of these miRNA biomarker candidates.

## Conclusions

Our results support the feasibility of quantifying miRNAs in serum samples as differentiators for PTB. More importantly, Simoa has great potential for miRNA detection owing to its high sensitivity and because it does not require pre-labelling, reverse transcription, or amplification. Simoa technology warrants further research in a large, well-characterised longitudinal patient cohort.

## Data Availability

The original contributions presented in the study are included in the article/**Supplementary Material**. Further inquiries can be directed to the corresponding author.
