# Supplementary material for: Single molecule measurements of microRNAs in the serum of patients with pulmonary tuberculosis
Source: Front Immunol. 2024 Sep 2;15:1418085. doi: 10.3389/fimmu.2024.1418085 (PMC11402676; doi:10.3389/fimmu.2024.1418085)
Supplement: Supplementary file 1 [file Table1.docx]

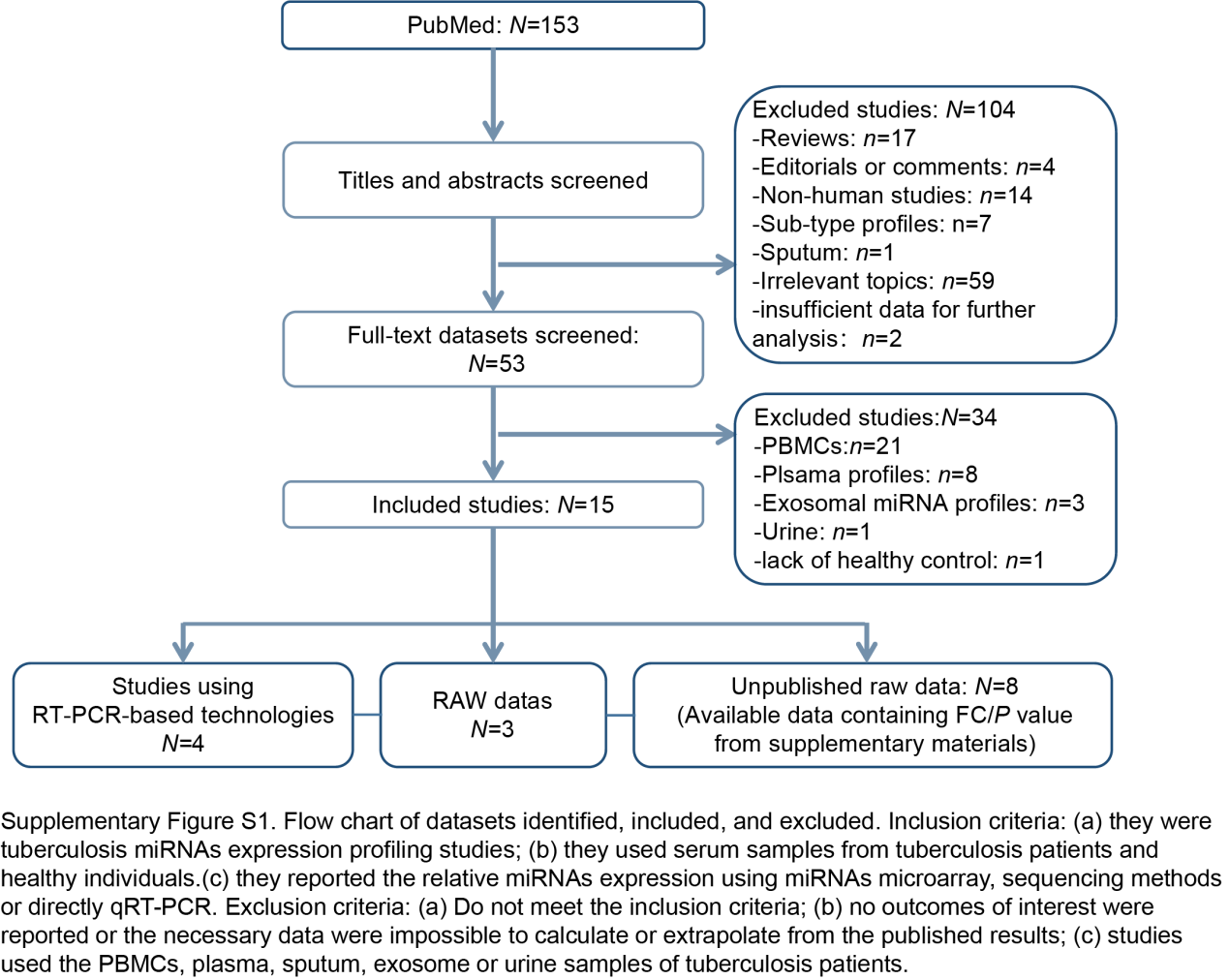


Supplementary Figure S1. Flow chart of datasets identified, included, and excluded. Inclusion criteria: (a) they were tuberculosis miRNAs expression profiling studies; (b) they used serum samples from tuberculosis patients and healthy individuals.(c) they reported the relative miRNAs expression using miRNAs microarray, sequencing methods or directly qRT-PCR. Exclusion criteria: (a) Do not meet the inclusion criteria; (b) no outcomes of interest were reported or the necessary data were impossible to calculate or extrapolate from the published results; (c) studies used the PBMCs, plasma, sputum, exosome or urine samples of tuberculosis patients.


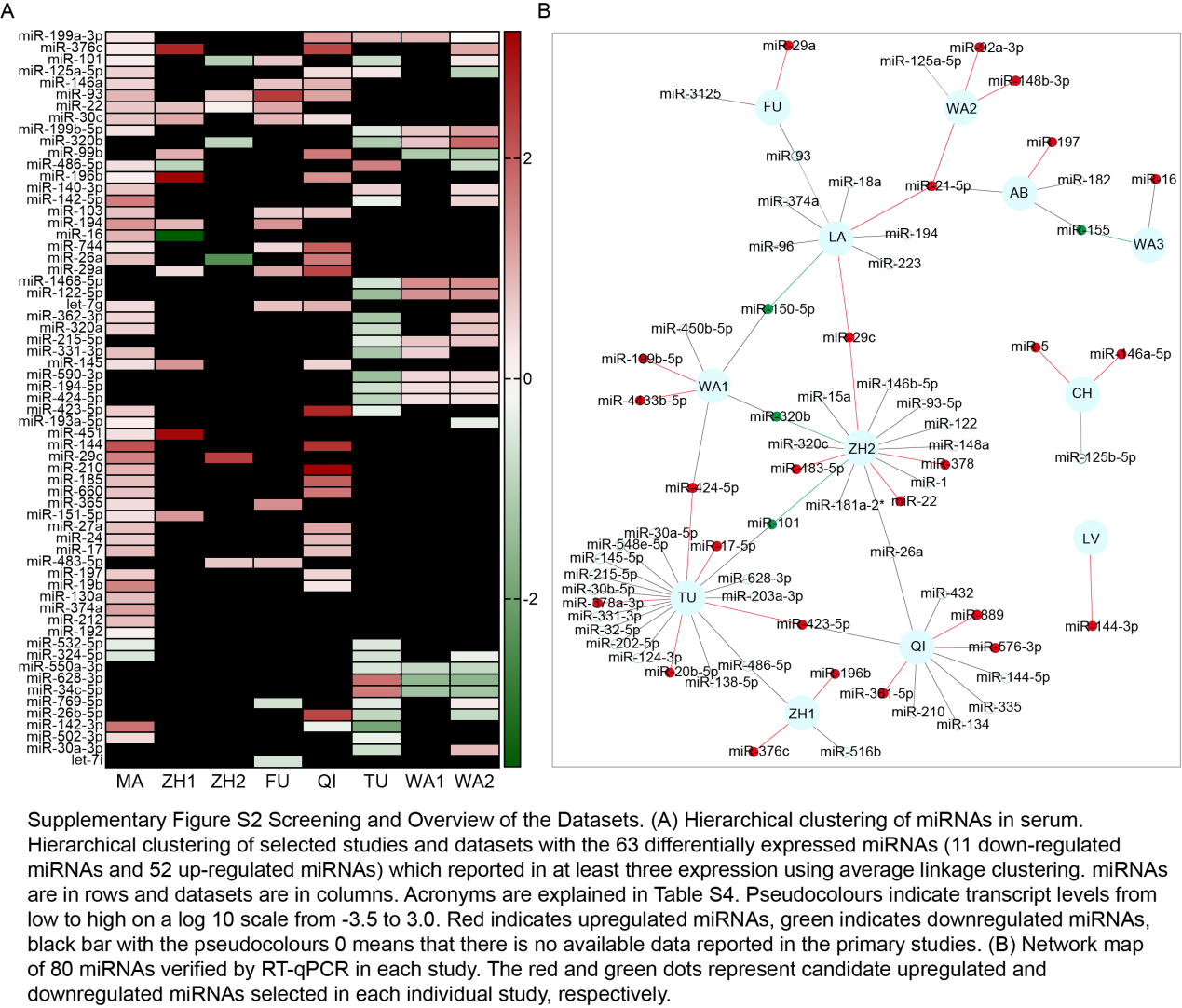


Supplementary Figure S2 Screening and Overview of the Datasets. (A) Hierarchical clustering of miRNAs in serum. Hierarchical clustering of selected studies and datasets with the 63 differentially expressed miRNAs (11 down-regulated miRNAs and 52 up-regulated miRNAs) which reported in at least three expression using average linkage clustering. miRNAs are in rows and datasets are in columns. Acronyms are explained in Table S4. Pseudocolours indicate transcript levels from low to high on a log 10 scale from -3.5 to 3.0. Red indicates upregulated miRNAs, green indicates downregulated miRNAs, black bar with the pseudocolours 0 means that there is no available data reported in the primary studies. (B) Network map of 80 miRNAs verified by RT-qPCR in each study. The red and green dots represent candidate upregulated and downregulated miRNAs selected in each individual study, respectively.
